# Advancing Cadmium Remediation via Rhizosphere Engineering: From Microbial Mechanisms to Field Deployment

**DOI:** 10.3390/plants15203152

**Published:** 2026-10-15

**Authors:** Teja Manda, Victor Edem Tsigbey, Kobina Kwansa Hammond, Adharsh Rajasekar, Raphael Dzinyela

**Affiliations:** 1 State Key Laboratory of Tree Genetics and Breeding, College of Biology and the Environment, Nanjing Forestry University, Nanjing 210037, China; teja.manda@njfu.edu.cn (T.M.); tsigbeyvictor@njfu.edu.cn (V.E.T.); hammondk.kwansa@njfu.edu.cn (K.K.H.); 2 Laboratory of Meteorological Disaster, Ministry of Education (KLME)/Joint International Research Laboratory of Climate and Environmental Change (ILCEC)/Collaborative Innovation Centre on Forecast and Evaluation of Meteorological Disasters (CIC-FEMD), Nanjing University of Information Science & Technology, Nanjing 210044, China; 3 School of Geography and Environmental Science, University of Reading, Reading RG67BE, UK; 4 Department of Chemistry and Biochemistry, Stephenson Life Sciences Research Center, University of Oklahoma, Norman, OK 73019, USA; raphael.dzinyela@ou.edu

**Keywords:** cadmium phytoremediation, rhizosphere engineering, rhizosphere microbiome, plant–microbe interactions, plant growth-promoting rhizobacteria

## Abstract

Cadmium (Cd) contamination of agricultural soils threatens food security and ecosystem health worldwide, yet conventional cleanup methods remain costly and ecologically damaging. Phytoremediation offers a sustainable in situ alternative, but low metal bioavailability, insufficient plant biomass, and inconsistent microbial performance limit field deployment. This review provides a framework that links molecular plant–microbe interactions to practical rhizosphere engineering for Cd management. We compare how functional traits of plant growth-promoting rhizobacteria (PGPR), arbuscular mycorrhizal fungi (AMF), and endophytes influence Cd mobilization versus immobilization, pH tolerance, and persistence. Their functions overlap and depend on plant species, Cd concentration, soil properties, and microbial strain; nevertheless, the evidence points to general tendencies: PGPR often drive short-term Cd mobilization with limited persistence, AMF commonly promote more durable immobilization through hyphal sequestration, and endophytes frequently modulate internal metal transport. We also evaluate synthetic consortia, multi-omics-guided microbiome design, and soil amendments. Field outcomes are appraised against explicit criteria (trial duration, replication across sites, use of historically contaminated soils, and reporting of Cd removal rate, translocation factor, available soil Cd, biomass, and soil health indicators), which also underpin the proposed standardized validation protocols. Matching microbial traits to site-specific goals, alongside food-safety, cost, and regulatory considerations, can help move phytoremediation toward scalable deployment work provides environmental managers and policymakers with a comprehensive roadmap for sustainable Cd remediation in agricultural landscapes.

## 1. Introduction

Cadmium (Cd) is a global toxic pollutant because it is transported long distances, persists, and accumulates biologically. Widespread anthropogenic release, persistence, and mobility of cadmium in soils, along with its uptake and translocation by plants, facilitate accumulation in edible plant tissues and subsequent transfer to the human food chain, posing a potential risk to food safety and human health [1]. Global anthropogenic Cd emissions to the environment are estimated at approximately 30,000 tonnes per year, with major sources including zinc-lead smelting, coal combustion, electroplating industries, municipal solid waste incineration, and, most pervasively in agricultural contexts, the application of phosphate fertilizers derived from Cd-rich phosphate rock deposits [1,2]. Irrigation with industrial wastewater and sewage sludge has created localized Cd hotspots in South and East Asia, parts of sub-Saharan Africa, and peri-urban agricultural zones across Europe and South America [3]. Yang Liu et al. conducted a field assessment in the lower Minjiang River basin and showed that cropping system design strongly influences cadmium removal from contaminated topsoil, with maize–sweet potato–cabbage rotations achieving the highest extraction efficiency. Although edible plant tissues consistently accumulate lower metal concentrations than non-edible parts, direct human consumption still exceeds established safety thresholds by nearly tenfold, driven primarily by vegetable intake. Diverting harvested biomass to livestock feed prior to human consumption could therefore mitigate dietary risks while preserving the soil remediation potential of these agricultural systems [4]. Cd causes severe carcinogenesis, respiratory harm, bone demineralization, amongst others in humans. Unlike organic contaminants, Cd is not biodegradable; it accumulates persistently in the plow layer of agricultural soils, with half-lives often exceeding decades under typical soil conditions. The lack of physiological excretion mechanisms results in a lifelong cumulative burden that surpasses that of many other toxic metals [5].

The geochemical behavior of Cd in soil is determined by a complex interplay of pH, organic matter content, clay mineralogy, competing cation concentration (particularly Zn^2+^ and Ca^2+^), and redox potential. The mobility and bioavailability of Cd are usually higher under acidic to moderately acidic soils due to the protonation of soil surfaces that decreases the binding of Cd and increases soil solution Cd concentrations. The ratio of free Cd^2+^ to organically and inorganically complexed Cd varies among soil types, depending on factors such as pH, organic matter content, clay content, cation-exchange capacity, and concentrations of Fe/Mn oxides [6]. Cd contamination significantly degrades soil health by disrupting biological and chemical properties, standing out among heavy metals primarily because of its exceptional mobility and high bioavailability. It disrupts microbial activities, including microbial biomass, and suppresses key soil enzymes; it also impairs nitrogen (N) mineralization and metabolism, lowering overall soil fertility and productivity. This has led to documented Cd accumulation in staple crops, including rice (*Oryza sativa*), wheat (*Triticum aestivum*), and leafy vegetables across regions where phosphate fertilization has been intensive for decades [7,8]. Conventional approaches to managing Cd-contaminated soils, including excavation and landfilling, soil washing, chemical immobilization, and electrokinetic remediation, face critical limitations in terms of cost, scalability, and ecological disruption [9]. Excavation and off-site disposal, while effective, can cost US$250,000–$1,000,000 per hectare and irreversibly destroy soil structure, microbial communities, and organic matter. In situ immobilization using lime, phosphate, or zeolite amendments can reduce Cd mobility but does not remove the metal from the site; residual contamination remains a long-term risk [10]. Furthermore, these approaches fail to address contamination heterogeneity across field scales. By contrast, phytoremediation exploits plants’ natural capacity to concentrate contaminants in harvestable biomass (phytoextraction), immobilize metals in the root zone (phytostabilization), or transform metal speciation (phytovolatilization), offering a low-input, in situ alternative compatible with continued land use [11]. Because Cd is an element that cannot be degraded, phytoremediation mobilizes, concentrates, or immobilizes it rather than destroying it. The underlying processes, including root exudation, rhizosphere acidification, and microbial Cd transformation, occur naturally in the rhizosphere of most plants, so some Cd attenuation takes place without external inputs; however, natural rates are usually too slow or inconsistent for practical cleanup, and plant selection and microbial inoculation are used to accelerate and stabilize these existing processes rather than to introduce new ones.

Despite its conceptual elegance, phytoremediation faces substantive biological constraints that limit practical deployment. Chief among these is the low phytoavailability of Cd in many soils, particularly those with circumneutral to alkaline pH, high clay content, or elevated organic matter, where Cd is extensively sorbed onto mineral surfaces or complexed with humic substances [12]. Even when Cd is mobilized, many commercially important crop species cannot translocate significant quantities from roots to shoots, a prerequisite for effective phytoextraction [13]. True Cd hyperaccumulator plants that accumulate >100 mg Cd kg^−1^ dry weight in aerial tissues are mostly confined to the Brassicaceae (e.g., *Thlaspi caerulescens*, *Noccaea caerulescens*) and certain Crassulaceae (e.g., *Sedum alfredii*). However, these typically produce modest biomass, limiting total Cd removal per growing season [14,15]. Additionally, Cd exerts direct phytotoxic effects at elevated concentrations, impairing photosynthesis, disrupting mineral nutrient uptake, inducing oxidative stress, and inhibiting root elongation, thereby reducing biomass production, which depends on it [16]. Phytoremediation therefore refers to the use of plants in general. However, capacity differs widely among species: both perennial and annual plants release root exudates and take up some Cd, whereas hyperaccumulators are rare. Many crops and high-biomass species accumulate less Cd per unit of tissue yet can remove comparable amounts through greater biomass.

The rhizosphere represents a biogeochemical hotspot where plant root exudates, comprising organic acids, amino acids, sugars, phenolics, and enzymes, create a chemically distinct zone that differs fundamentally from bulk soil in pH, redox potential, dissolved organic carbon content, and microbial community composition and activity [17]. This zone is the primary site of Cd mobilization, root uptake, and microbial transformation and therefore the most strategic target for engineering interventions to enhance phytoremediation efficiency [18]. The synergy between plant traits and rhizosphere microbiome functions, including microbial-driven changes in Cd speciation, the production of phytohormones and metal-chelating compounds, and modulation of plant stress tolerance, offers a multidimensional lever for overcoming the biological constraints that limit conventional phytoremediation. Moving beyond descriptive summaries of plant–microbe interactions, this review establishes a translational framework that connects molecular mechanisms to field-scale implementation. We critically evaluate emerging strategies, including synthetic consortia, multi-omics-guided microbiome design, and precision soil engineering, while addressing the laboratory-to-field gap, food safety trade-offs, regulatory pathways, and standardized validation protocols. By integrating mechanistic insights with practical deployment frameworks, this work provides an actionable roadmap for scaling rhizosphere-engineered phytoremediation toward real-world soil restoration.

## 2. Rhizosphere Processes Regulating Cd Mobility, Uptake, and Detoxification

### 2.1. Root Exudation and Chemical Modification of Cd Bioavailability

Both perennial and annual plants produce root exudates, but the composition is species-specific; they constitute a metabolically diverse and quantitatively significant flux of organic compounds from living roots into the rhizosphere, accounting for 20–40% of total net photosynthetically fixed carbon in some plant species [19]. In the context of Cd dynamics, low-molecular-weight organic acids, particularly citric, malic, oxalic, and tartaric acids, play a dual and context-dependent role [20]. At acidic pH, these compounds can chelate Cd^2+^ to form soluble organic complexes, effectively mobilizing Cd from mineral-bound pools and increasing its availability for root uptake. Conversely, in calcareous or near-neutral soils, organic acid-driven acidification of the immediate rhizosphere can transiently increase Cd^2+^ activity in the soil solution. Stable Cd-amino acid complexes, including histidine and nicotianamine, facilitate metal chelation in both the rhizosphere and the plant apoplast [21]. The chemical speciation resulting from root exudate–Cd interactions ultimately determines the fraction of Cd accessible to high-affinity root uptake transporters.

### 2.2. Rhizosphere pH, Redox Dynamics, and Soil Chemistry

Soil pH strongly controls Cd speciation and solubility, with Cd^2+^ activity in soil solution typically increasing 1.5–3-fold for each unit decrease in pH below 7.0. Proton extrusion by root plasma membrane H^+^-ATPases, coupled with the release of acidic root exudates and the respiratory production of CO_2_, creates a rhizosphere pH that can be 0.5–2.0 pH units lower than bulk soil in many plant species [22,23]. This acidification substantially mobilizes Cd from carbonate, phosphate, and clay-bound pools into the soil solution, where it becomes bioavailable for root uptake. However, rhizosphere acidification is not uniform: it varies among plant species, with nitrogen sources such as NH_4_^+^ vs. NO_3_^−^ strongly influencing proton balance, and rhizosphere microbial activity can amplify or buffer root-driven pH changes [24]. Redox dynamics are particularly important in waterlogged soils, such as paddy systems, where reducing conditions favor Cd precipitation as CdS, effectively immobilizing it; upon drainage and oxidation, Cd remobilizes, creating pulsed Cd availability events with major implications for rice grain contamination [25]. Engineering rhizosphere redox conditions, for example, through controlled water table management or targeted amendment applications, therefore represents a meaningful lever for managing Cd mobility in both phytoextraction and phytostabilization contexts: rhizosphere and cellular mechanisms regulating cadmium (Cd) uptake and detoxification in plants.

### 2.3. Root System Architecture and Cd Acquisition

**Figure 1:**
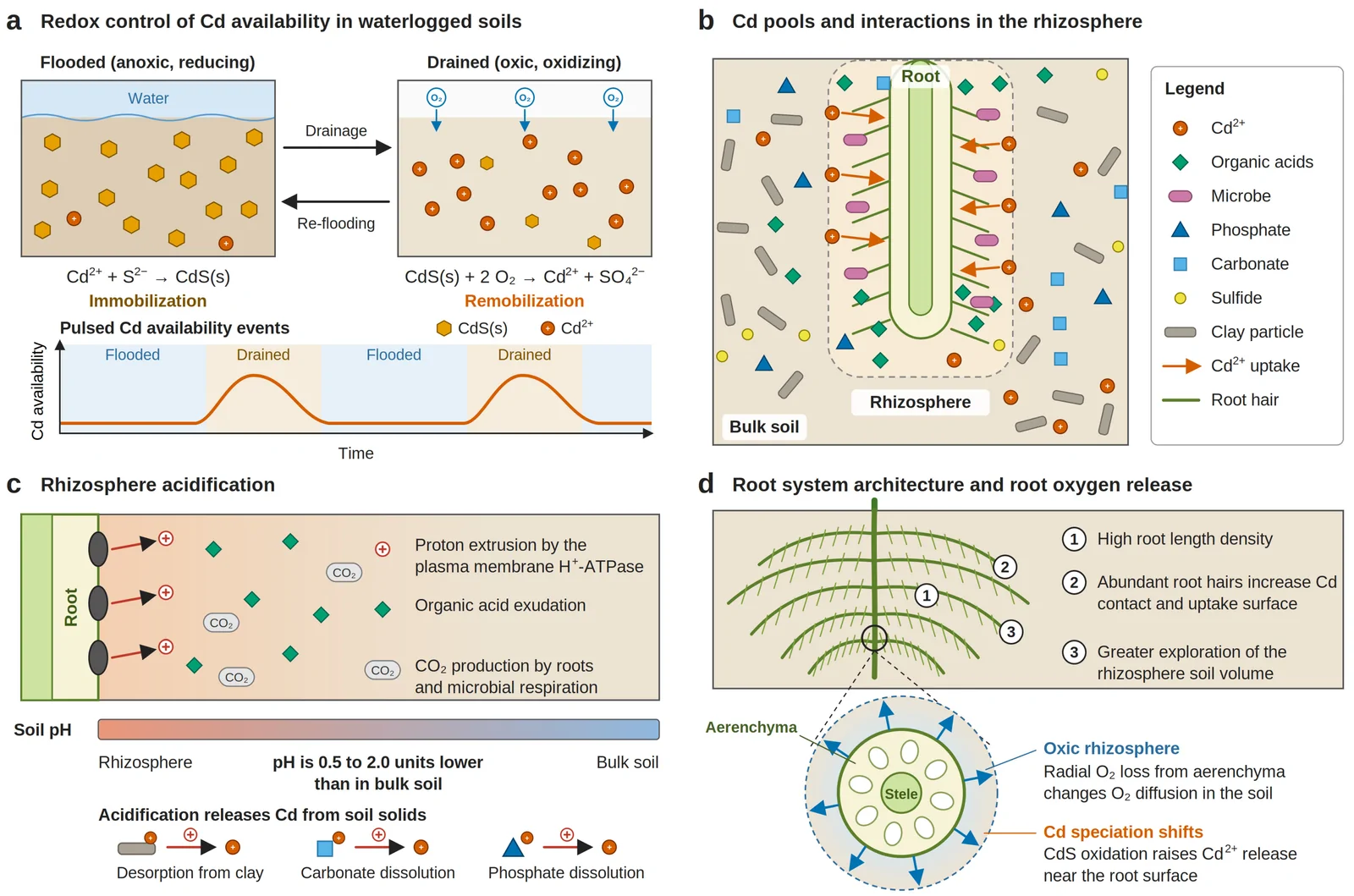
Rhizosphere physicochemical and root architectural controls on cadmium (Cd) bioavailability and acquisition. Teja Manda (2026) (https://app.biorender.com/illustrations/6ac7ceba5ad9434c1feead09?slideId=41c42a54-01d1-4607-a0f2-3fde38484422) (accessed on 15 September 2026).

Root system architecture (RSA), encompassing root length density, lateral root branching, root hair density and length, and root depth distribution, determines the physical extent of rhizosphere influence and the surface area available for Cd uptake [25]. Plants with high lateral root densities and abundant root hairs maximize contact with soil volumes where Cd is concentrated, and root hairs in particular can extend the effective uptake surface by up to tenfold. The Casparian strip in the endodermis constitutes a critical apoplastic barrier that routes Cd through the symplast for radial transport to the stele, while suberin lamellae in the exodermis provide an additional exclusion barrier in some species [26,27]. Aerenchyma formation under low oxygen stress, particularly in flood-tolerant species such as rice, alters radial oxygen loss patterns and Cd speciation in the immediate rhizosphere [28]. Understanding how Cd-induced changes in RSA, including root growth inhibition, cessation of root hair elongation, and increased lateral branching, feed back to alter Cd acquisition efficiency is critical for breeding and engineering strategies aimed at enhancing biomass production under metal stress. The low-level stimulatory effect does not mean that Cd has beneficial or nutritional value to plants but rather, it reflects a hormetic-type or stress-signaling response to low doses, and, in general, increases in Cd exposure are expected to result in inhibition of root growth and toxicity (Figure 1).

### 2.4. Plant Hormonal Crosstalk Under Cd Stress

**Figure 2:**
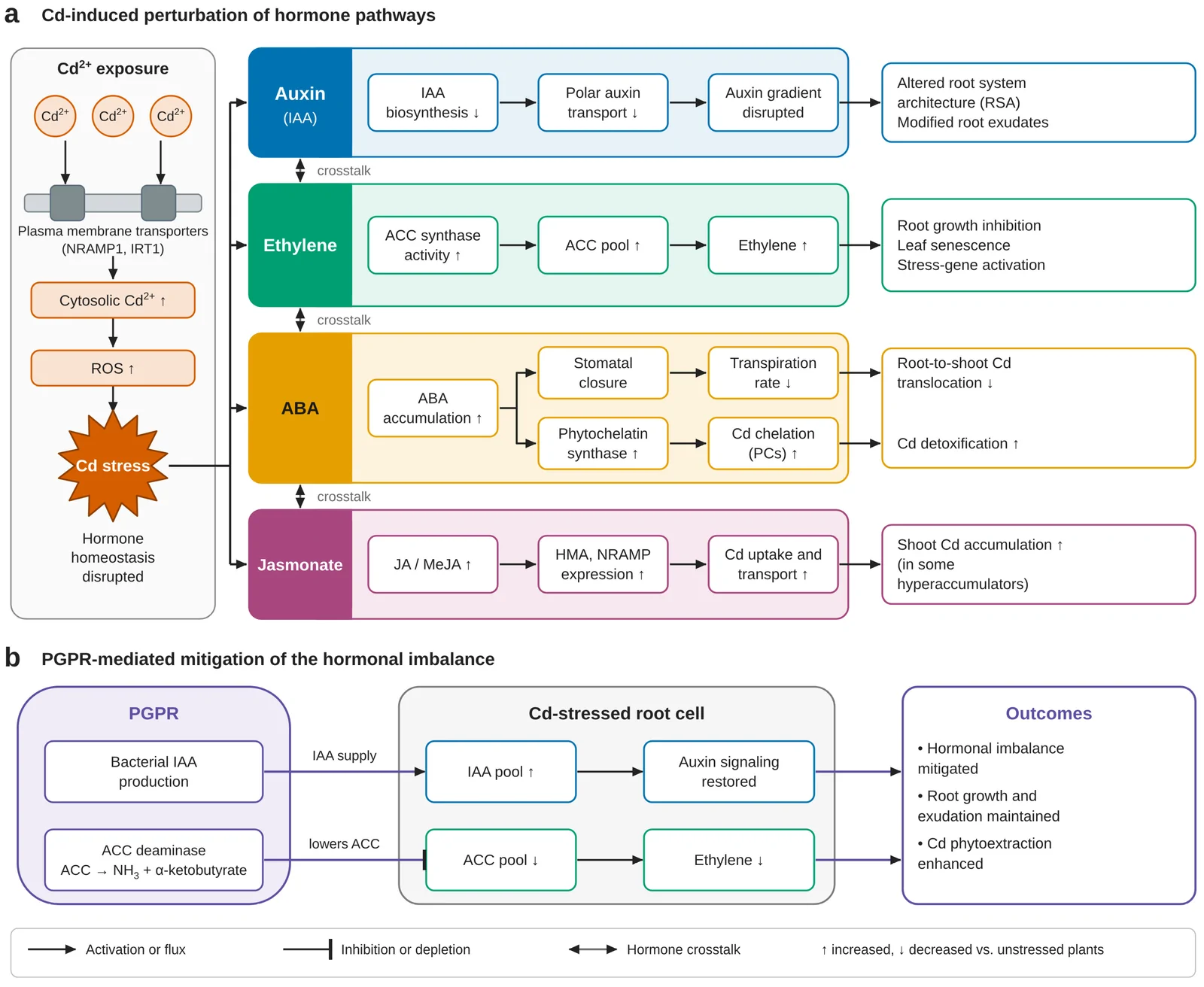
Cadmium perturbs auxin, ethylene, ABA, and jasmonate pathways, and PGPR counteract the imbalance. (**a**) Cd^2+^ enters root cells through plasma membrane transporters, raises cytosolic Cd^2+^ and ROS, and disturbs hormone homeostasis. Each hormone module shows the key changes and physiological outcomes. (**b**) PGPR supply bacterial IAA and degrade ACC through ACC deaminase, which restores auxin signaling, lowers ethylene, and maintains root growth and exudation, supporting Cd phytoextraction. Arrows indicate activation or flux, blunt-ended bars indicate inhibition or depletion, and double-headed arrows indicate crosstalk Teja Manda (2026) (https://app.biorender.com/illustrations/6ac7d4dba1907df051596737?slideId=8a509a80-a96c-4284-9dfe-b26ef60ab122) (accessed on 15 September 2026).

Plant hormonal networks undergo profound reorganization in response to Cd stress, with cascading effects on root system architecture, exudation chemistry, and tolerance responses. Cd can disrupt auxin (indole-3-acetic acid, IAA) signaling by inhibiting auxin biosynthesis enzymes and polar transport machinery, leading to altered root gravitropism, reduced lateral root initiation, and modified root exudate composition [29,30]. Cd also induces ethylene biosynthesis via ACC (1-aminocyclopropane-1-carboxylate) synthase activation, and elevated ethylene concentrations contribute to root growth inhibition, accelerated leaf senescence, and enhanced expression of stress-responsive genes, including metallothioneins [31]. Abscisic acid (ABA) accumulation under Cd stress modulates stomatal closure, reducing transpiration-driven Cd translocation to shoots while simultaneously inducing phytochelatin synthase expression [32]. Jasmonates play an emerging, complex role, and exogenous methyl jasmonate treatment has been shown to enhance Cd accumulation in the shoot tissues of certain hyperaccumulators by upregulating HMA (heavy metal ATPase) and NRAMP transporter expression [33]. Interestingly, plant growth–promoting rhizobacteria (PGPR) that produce IAA or ACC deaminase can partially counteract Cd-induced hormonal perturbations, thereby maintaining root growth and exudate output under metal stress, a mechanistic basis for their observed enhancement of Cd phytoextraction. These hormonal responses vary with different plant species, tissues, the concentration of Cd, exposure duration, as well as experimental conditions. The coordinated effects of Cd on auxin, ethylene, ABA, and jasmonate signaling, together with the capacity of PGPR to alleviate Cd-induced hormonal disruption, are summarized in Figure 2.

### 2.5. Plant Cellular Detoxification Pathways

**Figure 3:**
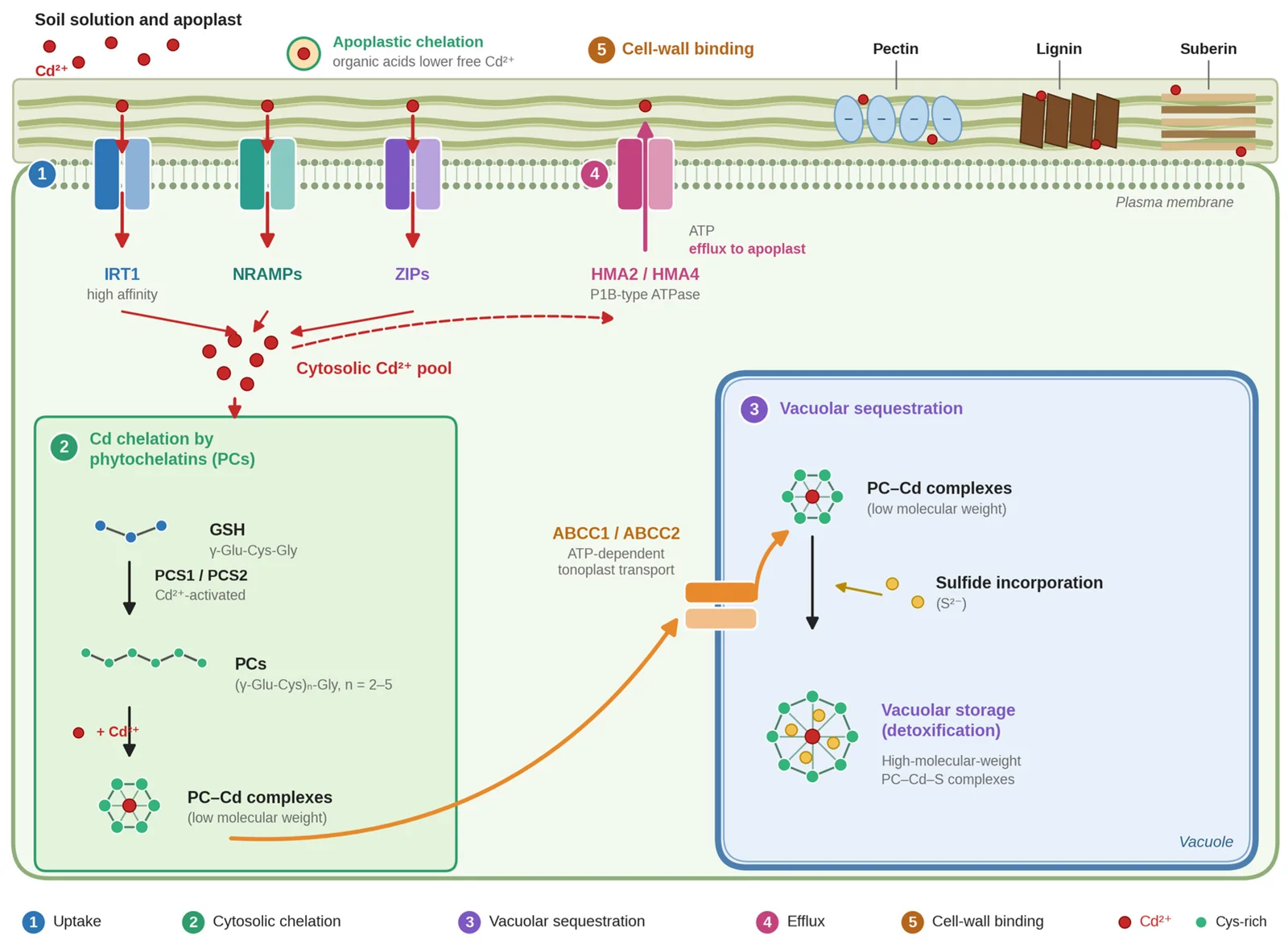
Cellular mechanisms of Cd uptake, chelation, sequestration, and efflux in a plant root cell. Cd^2+^ enters the cell through plasma-membrane transporters (IRT1, NRAMPs and ZIPs) (1). In the cytosol, Cd^2+^ is chelated by phytochelatins (PCs), which phytochelatin synthases (PCS1/PCS2) synthesize from glutathione (GSH) (2). The PC–Cd complexes are transported across the tonoplast by ABCC1/ABCC2 and sequestered in the vacuole, where sulfide incorporation produces high-molecular-weight PC–Cd–S complexes for detoxification (3). Excess Cd^2+^ is exported to the apoplast by the P1B-type ATPases HMA2 and HMA4 (4). In the apoplast, organic acids chelate Cd^2+^, and the cell wall (pectin, lignin, suberin) binds Cd, restricting its entry (5). ABCC, ATP-binding cassette subfamily C; GSH, glutathione; HMA, heavy metal ATPase; IRT1, iron-regulated transporter 1; NRAMP, natural resistance-associated macrophage protein; PC, phytochelatin; PCS, phytochelatin synthase; ZIP, ZRT/IRT-like protein. Teja Manda (2026) (https://app.biorender.com/illustrations/6acf13a2c5459bb269bbbd7c?slideId=0f44a443-a61a-43d4-9c91-ad9ef5239b07) (accessed on 15 September 2026).

Once Cd enters root cells, a coordinated network of molecular mechanisms drives its detoxification and sequestration. After cellular uptake, coordinated chelation, vacuolar sequestration, cell-wall binding, and transporter-mediated efflux pathways constrain Cd toxicity, as summarized in Figure 3. Phytochelatins (PCs), enzymatically synthesized thiol-rich peptides derived from glutathione by phytochelatin synthases (PCS1/PCS2), represent the primary mechanism of Cd chelation in most plant species. PC–Cd complexes are transported into the vacuole by ABCC-type transporters (e.g., AtABCC1 and AtABCC2 in Arabidopsis), where they form high-molecular-weight complexes stabilized by sulfide incorporation [34,35].

Metallothioneins (MTs), small cysteine-rich proteins encoded by MT gene families, provide complementary Cd binding capacity, particularly in root epidermal cells and during seed development [36]. In parallel, Cd exposure induces a rapid and large-scale oxidative burst, with reactive oxygen species (ROS) produced at the plasma membrane, chloroplasts, and mitochondria; the enzymatic antioxidant network comprising superoxide dismutase (SOD) [37], ascorbate peroxidase (APX), catalase (CAT), and glutathione reductase (GR) is upregulated in tolerant genotypes to buffer ROS accumulation and prevent irreversible oxidative damage to membranes, DNA, and proteins [38]. The balance between PC-mediated Cd chelation and vacuolar sequestration in roots versus long-distance xylem loading and translocation to shoots is determined by the relative activity and expression of competing HMA and NRAMP transporters. It represents a critical engineering target for enhanced phytoextraction [39].

## 3. Plant–Microbe Interactions Enhancing Cd Tolerance and Phytoextraction

### 3.1. Plant Growth-Promoting Rhizobacteria (PGPR)

PGPR are a functionally diverse assemblage of rhizosphere bacteria that enhance plant growth and stress tolerance through multiple direct and indirect mechanisms [40]. In Cd remediation, three PGPR-mediated mechanisms are particularly relevant. First, siderophores, particularly the fluorescent siderophores produced by Pseudomonas spp. and the catecholate-type siderophores produced by Bacillus spp., are primarily produced for Fe^2+^ acquisition but can sometimes form stable complexes with Cd, altering its speciation and mobility in the rhizosphere [41]. Direct Cd complexation has been demonstrated for specific siderophores such as pyochelin. After the stable complex has formed, Cd can either dissolve and be mobilized, hence increasing cadmium’s mobility, making it easier to wash away or move through the rhizosphere, or be immobilized since many siderophore-metal complexes bind to soil particles, organic matter, or mineral surfaces, thus locking up Cd and preventing it from either being absorbed by plants or leached into the groundwater. Second, ACC deaminase-producing PGPR can break down the ethylene precursor 1-aminocyclopropane-1-carboxylate (ACC), helping reduce Cd-induced ethylene production and the inhibition of root growth. These bacteria can maintain root development and physiological activity under Cd stress, helping plants take up more Cd and maintain rhizosphere interactions, leading to higher harvestable Cd accumulation. Therefore, using PGPR to maintain plant health could improve phytoextraction efficiency by increasing Cd uptake from contaminated soil and increasing Cd accumulation in harvested plant biomass [42]. Third, exopolysaccharide (EPS)-producing strains such as *Bacillus subtilis* and *Pseudomonas aeruginosa* form biofilms on root surfaces that can directly biosorb Cd^2+^ through carboxyl, hydroxyl, and amino functional groups in the EPS matrix, immobilizing Cd at the root surface and modulating the concentration of free Cd^2+^ in the rhizosphere [43]. Co-inoculation experiments with strains combining ACC deaminase and siderophore activities have demonstrated synergistic effects on both plant biomass and shoot Cd accumulation, with translocation factors exceeding those of single-strain inoculations by up to 65% in controlled experiments (Table 1).

### 3.2. Mycorrhizal Fungi and Cd Stress Mitigation

Arbuscular mycorrhizal fungi (AMF) form obligate symbioses with most terrestrial plant species, extending the root–soil interface through an extensive extraradical mycelium that can explore soil volumes inaccessible to roots. In Cd-contaminated soils, AMF play a dual role that varies by context [50]. At low-to-moderate Cd concentrations, AMF colonization enhances plant growth through improved phosphorus, zinc, and micronutrient acquisition, and this nutritional benefit partially offsets Cd-induced toxicity [51]. AMF hyphae produce glomalin-related soil proteins (GRSPs), glycoproteins that can bind significant quantities of Cd, Cu, Pb, and other metals in the hyphal wall and in soil aggregates, effectively sequestering Cd away from root uptake sites [52]. Within root cortical cells, AMF arbuscules modulate the plant’s metal-homeostasis machinery. Colonization by *Rhizophagus irregularis* (formerly *Glomus intraradices*) downregulates plant ZIP- and IRT-family Cd uptake transporters while upregulating vacuolar sequestration pathways, reducing Cd translocation to shoots [53]. This immobilization function can be advantageously exploited in phytostabilization strategies, while targeted selection of AMF strains that do not restrict shoot translocation is critical for phytoextraction applications.

### 3.3. Endophytic Bacteria and Fungi

Endophytic microorganisms, including bacteria and fungi that colonize internal plant tissues without causing disease symptoms, represent an often underappreciated component of plant–microbe interactions relevant to Cd remediation [54]. Endophytic bacteria, including *Burkholderia cepacia*, *Methylobacterium oryzae*, and various *Bacillus* spp. inhabit root cortex, vascular tissue, and stem parenchyma, where they can produce IAA, cytokinin, and ACC deaminase at sites inaccessible to rhizosphere PGPR [55]. Critically, endophytes can modulate the expression of plant metal transporter genes, including HMA4, NRAMP3, and ZIP4, within plant tissue, thereby directly influencing Cd partitioning between roots and shoots. Endophytic fungi, particularly dark septate endophytes and certain Trichoderma species, have been documented to upregulate phytochelatin synthase expression and enhance vacuolar Cd sequestration, increasing plant tolerance to concentrations that would otherwise be growth-limiting [56]. The stability of endophytic colonization under field conditions, and the degree to which inoculated strains can competitively establish within host plant tissues alongside native endophyte communities, remain active areas of investigation.

### 3.4. Microbial Genes and Enzymatic Pathways in Cd Transformation

Microbial communities in Cd-contaminated rhizospheres harbor extensive genetic machinery for metal transformation, bioaccumulation, and resistance. Cd-resistant bacteria typically carry chromosomal or plasmid-borne determinants including czc (Cd/Zn/Co efflux), cad (Cd-specific P-type ATPase efflux), and mer-associated genes that confer tolerance via active efflux, intracellular sequestration, or metal precipitation as CdS using sulfate-reducing bacteria [57]. Metagenomic surveys of hyperaccumulator rhizospheres, such as those of *Sedum alfredii* and *Noccaea caerulescens*, have revealed significant enrichment of microbial taxa encoding organic acid biosynthesis genes and metal efflux pumps relative to non-accumulator rhizospheres. This microbial community signature may contribute to the bioavailability-enhancing rhizosphere chemistry characteristic of hyperaccumulators [58]. Sulfate-reducing bacteria (SRB) are particularly interesting for engineered systems: their H_2_S production drives CdS nanoparticle precipitation, immobilizing Cd in a minimally bioavailable form [59]. Interestingly, sulfur-oxidizing bacteria can remobilize CdS under oxic conditions, underscoring the importance of understanding microbial community dynamics and redox regimes for effective rhizosphere management.

### 3.5. Case Studies of Microbe-Assisted Cd Remediation

The most extensively documented natural Cd hyperaccumulator systems provide critical benchmarks for rhizosphere engineering strategies. *Thlaspi caerulescens* (now *Noccaea caerulescens*), capable of accumulating >10,000 mg Cd kg^−1^ dry weight in shoots, maintains a distinct rhizosphere microbiome enriched in Cd-mobilizing taxa; however, its low biomass (typically <1 t dry matter ha^−1^ yr^−1^) severely limits absolute Cd removal [60]. *Sedum alfredii*, a Chinese hyperaccumulator with substantially higher biomass and comparable Cd accumulation capacity, has been the subject of rhizosphere microbiome characterization studies that identified key PGPR taxa (notably *Pseudomonas* and *Burkholderia* spp.) as consistent components of the hyperaccumulator rhizosphere [61]. Field-scale trials combining *Sedum alfredii* with PGPR inoculation in China have achieved Cd removal rates of 60–90 g ha^−1^ season^−1^, representing a 2–3-fold improvement over uninoculated controls. In crop-based systems, inoculation of *Zea mays* with *Glomus mosseae* has improved plant tolerance and nutrient status while modulating Cd shoot concentrations, though the direction of effect on Cd translocation depends critically on AMF strain selection [62].

## 4. Rhizosphere Engineering Strategies to Enhance Cd Phytoremediation

### 4.1. Microbial Inoculant Design and Synthetic Consortia

Designing effective microbial inoculants for Cd phytoremediation requires moving beyond single-strain applications toward rationally assembled synthetic consortia that combine complementary functional traits [63]. Successful co-inoculation strategies pairing ACC deaminase-producing strains with siderophore-producing bacteria have produced greater plant growth or metal-uptake responses that exceed the sum of individual-strain contributions reported in Table 2. A critical but frequently neglected consideration in consortium design is inter-strain compatibility. Some PGPR produce antibiotics or quorum-sensing inhibitors that suppress coinoculant populations, undermining consortium function in vivo [64]. Biofilm-forming inoculants that colonize seed surfaces or root mucilage persist longer in the competitive rhizosphere, and encapsulation in alginate or κ-carrageenan matrices has been shown to extend inoculant shelf life and survival in field soils [65]. Emerging approaches include engineering synthetic gene circuits into PGPR strains to enable inducible, Cd-responsive production of siderophores or exopolysaccharides, triggered specifically under elevated soil Cd conditions and coupling inoculant activity to the remediation target [66].

### 4.2. Soil Amendments and Their Effects on Cd Dynamics

**Figure 4:**
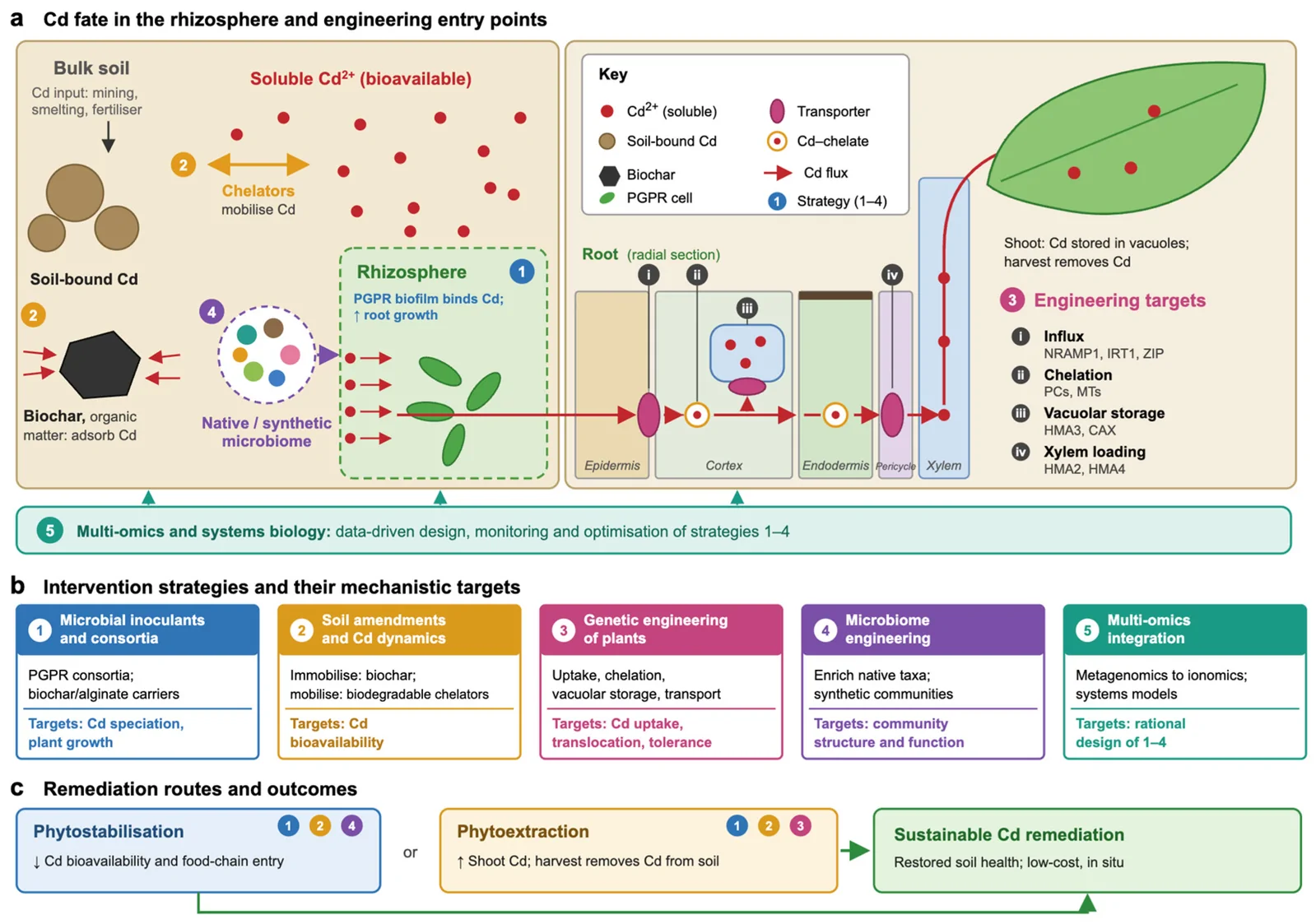
Strategies for engineering cadmium (Cd) fate in the soil–rhizosphere–plant system. (**a**) Cd entering soil from anthropogenic inputs partitions between soil-bound and soluble, bioavailable Cd^2+^. Soil amendments (2), such as biochar, immobilize Cd, whereas chelators mobilize it. In the rhizosphere, plant growth-promoting rhizobacteria (PGPR; 1) and native or synthetic microbiomes (4) modify Cd speciation and root growth. Within the root (radial section), Cd moves through the epidermis, cortex, endodermis, and pericycle into the xylem and then to the shoot. Four plant engineering targets (3) are indicated: (i) influx, (ii) chelation, (iii) vacuolar storage, and (iv) xylem loading. Multi-omics and systems biology (5) inform the design and optimization of strategies 1–4. (**b**) Intervention strategies 1–5 and the mechanistic targets of each. (**c**) Phytostabilization reduces Cd bioavailability and entry into the food chain, whereas phytoextraction removes Cd by harvesting shoot biomass. Colored numbers indicate the main strategies for each route. Both routes aim at sustainable Cd remediation. PCs, phytochelatins; MTs, metallothioneins. Teja Manda (2026) (https://app.biorender.com/illustrations/6acf1724d7135738fe5e48c2?slideId=cd5591f3-c614-4bc0-9250-9000f05b3221) (accessed on 15 September 2026).

Soil amendments represent a pragmatic, readily implementable tool for modulating rhizosphere Cd dynamics to support either phytoextraction (through Cd mobilization) or phytostabilization (through Cd immobilization) objectives. Biochar, a pyrogenically derived carbonaceous material produced from a wide range of organic feedstocks, has received substantial attention as a Cd immobilizer because of its high specific surface area (typically 100–500 m^2^ g^−1^), abundant surface functional groups, and liming effect that raises soil pH [18,73]. However, the magnitude of Cd sorption by biochar is strongly feedstock-dependent: biochar produced from poultry manure and sewage sludge at high temperatures (>600 °C) consistently outperforms wood-derived biochar in Cd sorption capacity [74]. Importantly, biochar amendments simultaneously enhance rhizosphere microbial biomass, activity, and diversity, creating a more favorable microbial habitat that can amplify PGPR inoculant effectiveness. For phytoextraction applications, synthetic chelators such as EDTA and EDDS (ethylenediamine-N, N′-disuccinic acid) can mobilize Cd 3–10-fold in calcareous soils by competing with soil minerals for Cd complexation, increasing shoot Cd accumulation substantially [75]. The primary concern with EDTA application is its resistance to biodegradation, which can drive chelated Cd leaching below the rooting zone into groundwater; biodegradable chelators, including citric acid, nitrilotriacetic acid (NTA) [76], and humic substances, offer a more environmentally responsible alternative, though with reduced mobilization efficacy shown in Figure 4.

### 4.3. Genetic Engineering of Plants for Improved Cd Uptake and Tolerance

Molecular genetic approaches to improving phytoremediation efficiency have focused primarily on three targets: enhancing root-to-shoot Cd translocation, increasing vacuolar sequestration capacity in shoots, and reducing Cd-induced phytotoxicity to sustain biomass production under metal stress [77]. Overexpression of HMA4, a P_1_B-type ATPase that loads Cd into xylem from root stele cells in *Arabidopsis thaliana* and *Nicotiana tabacum*, has produced 2–5-fold increases in shoot Cd concentration, confirming HMA4 as a primary rate-limiting step in Cd translocation [78,79]. In contrast, overexpression of OsHMA3 in rice, which encodes a tonoplast-localized Cd transporter directing Cd into root vacuoles, reduces grain Cd but also limits shoot accumulation, the opposite of what phytoextraction requires, illustrating that, in the direction of transporter engineering, the desirable transporter phenotype depends on the remediation objective [80]. Overexpression of phytochelatin synthase (AtPCS1) or metallothionein genes (MT2a, MT2b) has improved Cd tolerance and root accumulation, but does not necessarily result in increases in shoot translocation [81]. Combinatorial transgenic approaches simultaneously modifying uptake, translocation, and sequestration represent the frontier of plant engineering for phytoremediation.

### 4.4. Microbiome Engineering and Community-Level Manipulation

Rather than adding exogenous microbial strains, an alternative paradigm manipulates the indigenous rhizosphere microbiome to enrich beneficial taxa and suppress detrimental ones. Researchers can achieve selective enrichment through targeted soil amendments (organic carbon sources that favor specific functional guilds), intercropping with hyperaccumulator species that exude Cd-mobilizing compounds, or sequential application of PGPR inoculants that restructure community composition through competitive exclusion [82,83]. Rhizosphere microbiome transplantation, inoculating non-hyperaccumulator plants with rhizosphere soil or microbiome extracts from hyperaccumulator species, has shown promise in enhancing Cd mobilization and plant uptake, similar to transplanting disease-suppressive microbiomes in soil-borne pathogen contexts [84]. The specificity and durability of microbiome restructuring effects, particularly under variable field conditions, represent major knowledge gaps that must be addressed before these approaches can be reliably deployed [85]. Synthetic ecology approaches, in which researchers assemble defined synthetic communities of 5–20 well-characterized strains to recapitulate key functional properties of hyperaccumulator rhizosphere microbiomes, offer a more tractable path to mechanistic understanding and reproducible outcomes [86].

### 4.5. Integration of Multi-Omics for Targeted Rhizosphere Manipulation

Integrating multi-omics platforms has transformed our ability to characterize rhizosphere community structure and function mechanistically, enabling the identification of specific microbial taxa, genes, and metabolites that drive Cd mobilization, tolerance, and accumulation [87]. Metagenomic sequencing of rhizosphere DNA provides comprehensive inventories of microbial community membership and the community’s metabolic potential, including gene abundance for Cd efflux pumps, siderophore biosynthesis, ACC deaminase, and phosphate solubilization [57]. Metatranscriptomics sequencing of rhizosphere community RNA reveals which of these functions are actually expressed under field Cd stress conditions, overcoming the ‘metabolic potential vs. realized activity’ limitation of metagenomics [88]. Metabolomics profiling of rhizosphere soil solution by high-resolution mass spectrometry enables identification of the specific organic acid, amino acid, and phenolic compounds that characterize hyperaccumulator rhizospheres [89]. Ionomics high-throughput elemental analysis of plant tissues links shoot metal profiles to specific combinations of rhizosphere treatments and genetic backgrounds. Systems biology integration of these data layers, using network inference and constraint-based metabolic modeling, has begun to identify key regulatory nodes, such as specific PGPR taxa whose abundance correlates most strongly with the plant Cd translocation factor, as targets for microbiome engineering interventions [90].

## 5. Integrated Plant–Microbe Systems

### 5.1. Case Studies

Coupling hyperaccumulator plants with a selective pool of microbial partners is no longer a matter of proof of concept but of producing strong evidence at the pot, mesocosm, and field levels. *Sedum alfredii* Hance, a native Zn/Cd co-hyperaccumulator of southern China, is one of the best-studied model systems. Liu et al. [91] showed that inoculation of *S. alfredii* rhizospheres with a multi-metal-resistant Bacillus subtilis strain PY79 led to 88.02% in shoot Cd accumulation, as well as 58.99% in Zn, 90.22% in Cu, and 54.97% in Pb after a 30-day exposure in multi-metal-contaminated soils. These gains were attributed to rhizosphere acidification, siderophore production, and IAA-mediated root proliferation, all of which increased the metal-acquisition surface area and the bioavailable metal pool.

Also, in 2024, Huang et al. [92] conducted a 2-year field-sampling campaign across multiple contaminated sites to characterize the core endophytic microbiome of *S. alfredii*. Based on network analysis of 16S rRNA amplicon data, the authors assembled 97 core bacterial taxa into an optimized synthetic community (SynCom-NS). *Leifsonia shinshuensis*, *Novosphingobium lindaniclasticum*, *Ochrobactrum anthropi*, and *Pseudomonas izuensis* were among the taxa. Inoculation with SynCom-NS significantly upregulated genes associated with Cd transport, antioxidant defense, and phytohormone signaling in both hyperaccumulating and non-hyperaccumulating S. alfredii ecotypes, thereby promoting plant growth, Cd uptake, and above-ground translocation. A landmark multi-site field study by Cao et al. [93] assembled a four-strain consortium comprising *Sphingomonas* sp. SaMR12, *Burkholderia* sp. SaZR4, *Variovorax* sp. SaNR1, and *Burkholderia* sp. SaMR10 and applied it alongside *S. alfredii* across three geographically distinct contaminated sites in China. The multi-strain inoculation increased shoot dry weight by 3.79–22.9% relative to single-strain controls and enhanced shoot Cd accumulation to varying but consistently positive degrees across all three sites, underscoring the environmental adaptability advantages of consortium-based approaches. The results show that intercropping and inoculation, when combined, offer a viable approach to enhance Cd phytoextraction efficiency, amplifying plant biomass and metal uptake beyond additive effects. Similarly, the study on phytohormone-assisted phytoextraction shows a complementary trajectory. In 2022, Chen et al. reported that foliar spray of plant-growth-promoting exopolysaccharides (PGPE) enhanced *S. alfredii* biomass by 42.9% and Cd removal efficiency by 103.4%. When these exopolysaccharide treatments were combined with 0.2 mg/L indole-3-acetic acid (IAA) and 0.2 mg/L abscisic acid (ABA), shoot Cd phytoextraction increased relative to untreated controls. These results indicate that foliar application of IAA, BR, or ABA+BR can enhance *S. alfredii*’s phytoremediation efficacy in contaminated soil by increasing biomass and metal accumulation in harvestable portions.

### 5.2. Synergistic Effects on Cd Uptake and Tolerance

Mechanistically, the synergistic returns observed in case studies result from multiple interacting processes. Plant growth-promoting rhizobacteria (PGPR) and endophytes can simultaneously solubilize soil-bound Cd through organic acid production and proton extrusion, enlarging the labile Cd pool accessible to roots; synthesize IAA and gibberellins that expand root architecture, increasing the soil volume explored; produce ACC deaminase that lowers ethylene-mediated stress inhibition, sustaining plant growth under otherwise toxic Cd concentrations; and upregulate plant metal transporter genes (IRT1, HMA3, ZIP family) that mediate cellular uptake and vacuolar sequestration [94,95]. At the community scale, PGPR inoculation restructured the native rhizosphere microbiome, increasing remediation beyond that of the inoculant alone. Chi et al. [96] showed that effective PGPR colonization elevated soil nitrate reductase activity, driving a shift in the nitrogen-cycle microenvironment of the rhizosphere, which in turn enriched beneficial rare taxa, particularly *Lysobacter* and *Microbacterium* that act as “microbial partners” for the inoculant, fostering its persistence and Cd-mobilizing capacity. Another dimension to synergy includes the relationship between Arbuscular mycorrhizal fungi (AMF) and Cd remediation.

AMF colonization mitigates Cd-induced oxidative stress by enhancing catalase and superoxide dismutase activities, stabilizing photosystem II efficiency, and modulating plant phytochelatin and glutathione pools [97]. Research by Liu et al. [98] demonstrated that the combination of AMF-Selenium (Se) in fertilization increased the biomass of wheat under Cd stress while simultaneously shaping the composition of the rhizosphere microbial community, hence showing that the effects of AMF are partially mediated via downstream microbiome changes rather than direct plant physiology alone. Intercropping adds another dimension of plant–microbe synergy with applied relevance. Cao et al. [93] demonstrated that intercropping *Brassica napus* with *S. alfredii*, in addition to consortium inoculation, created a technical model for the simultaneous production and remediation of soils that are lightly to moderately contaminated with Cd. This offers a pathway to maintain farm income during long remediation cycles.

## 6. Future Perspectives, Challenges, and Research Opportunities

### 6.1. Field-Scale Validation: Critical Appraisal and Implementation Framework

#### 6.1.1. The Laboratory-to-Field Discrepancy

Despite mechanistic advances in rhizosphere engineering, a persistent laboratory-to-field gap continues to constrain the translation of Cd remediation from proof-of-concept to operational deployment. Most published efficacy data come from pot or greenhouse trials using artificially Cd-spiked soils, which systematically overestimate metal bioavailability, ignore historical aging effects, and oversimplify rhizosphere microbial complexity [99]. In historically contaminated field soils, Cd progressively sequesters into recalcitrant mineral phases, becomes occluded within organic matter matrices, or adsorbs to clay edges, reducing the labile fraction available for plant uptake by orders of magnitude compared with freshly spiked systems [100]. Furthermore, controlled environments lack the spatial heterogeneity, climatic variability, and competitive soil food webs that in situ dictate inoculant survival, amendment efficacy, and root exudation dynamics. Consequently, engineering strategies that show 2–5-fold increases in shoot Cd accumulation under optimal pot conditions often collapse to <50% efficacy or show high site-specific variability when deployed at the field scale [101].

#### 6.1.2. Critical Evaluation of Existing Field Evidence

Recent multi-site field trials provide valuable but sobering benchmarks for rhizosphere engineering. The landmark study by [102,103] demonstrated that a four-strain PGPR consortium applied alongside *Sedum alfredii* increased shoot dry weight by 3.79–22.9% across three geographically distinct contaminated sites, confirming that consortium-based approaches can buffer environmental variability better than single-strain inoculants. However, critical appraisal reveals consistent limitations: (i) most “field” studies span only 1–2 growing seasons, insufficient to capture seasonal rhizosphere turnover or long-term soil health trajectories; (ii) reporting lacks standardization, with inconsistent use of metrics such as Cd removal rate (g ha^−1^ yr^−1^), translocation factor shifts, or baseline microbiome recovery; and (iii) few trials integrate food safety monitoring, leaving unresolved the risk that mobilization strategies inadvertently increase Cd entry into adjacent food crops or groundwater [102]. While intercropping designs (e.g., *Brassica napus* + *S. alfredii*) offer promising agronomic benefits, researchers have not validated their economic and regulatory scalability beyond pilot plots.

#### 6.1.3. Temporal Dynamics, Microbial Persistence, and Long-Term Soil Health

A critical bottleneck in field deployment is the transient nature of engineered rhizosphere functions. PGPR inoculants typically colonize the rhizosphere rapidly, then decline competitively within 4–12 weeks as indigenous microbiota reassert dominance, nutrient pulses dissipate, or seasonal moisture/temperature shifts alter community assembly trajectories [103]. Biochar amendments persist longer but can drive unintended long-term shifts in soil pH, cation exchange capacity, and microbial guild ratios, particularly when feedstock composition or application rates are not site-matched [104]. Chelator-assisted phytoextraction yields immediate gains in Cd mobilization but poses documented risks of deep leaching, soil structure degradation, and dependence on repeated applications, raising sustainability concerns for multi-year remediation cycles [105]. Critically, few field studies track whether engineered traits (e.g., enhanced siderophore production, ACC deaminase activity, or transporter upregulation) are self-sustaining or require continuous re-inoculation. Longitudinal monitoring (>3–5 growing seasons) is essential to distinguish transient stimulation from stable rhizosphere restructuring, and to assess downstream impacts on soil aggregate stability, nitrogen cycling, and pathogen suppression [106].

#### 6.1.4. Toward Standardized Field Validation Protocols

To bridge the mechanistic-to-applied divide, the field must adopt standardized, multi-tiered validation frameworks. Recommended protocols should include: (1) baseline site characterization detailing Cd speciation (exchangeable, reducible, oxidizable, residual fractions), soil physicochemical properties, and native microbiome composition via amplicon/metagenomic sequencing; (2) randomized block designs replicated across at least three climatic/edaphic zones to capture genotype × environment × microbiome (G×E×M) interactions; (3) harmonized performance metrics, including annual Cd extraction efficiency (g ha^−1^ yr^−1^), translocation factor, soil available Cd (DTPA or CaCl_2_-extractable), plant biomass partitioning, and standardized soil health indicators (e.g., dehydrogenase activity, aggregate stability, microbial biomass carbon) [107]; and (4) longitudinal multi-omics tracking integrating seasonal metatranscriptomics, rhizosphere metabolomics, and ionomics to link microbial functional expression with plant Cd uptake phenotypes [108]. Integrate emerging sensor technologies, including portable XRF for spatial Cd mapping, drone-based multispectral imaging for biomass/stress monitoring, and Cd-responsive microbial biosensors, to enable precision application of inoculants and amendments at bioavailability hotspots. Open data sharing and standardized reporting templates will be critical to enabling cross-study meta-analyses and accelerating regulatory acceptance [109].

#### 6.1.5. Economic Viability and Regulatory Pathways

Field validation must be coupled with rigorous techno-economic analysis to assess real-world feasibility. While conventional physicochemical remediation can cost US$250,000–$1,000,000 ha^−1^ and permanently disrupt soil ecology [110], phytoremediation offers a low-input alternative but requires multi-year land dedication, repeated biomass harvests, and safe disposal or valorization of metal-laden plant material [111]. Cost–benefit models should account for input expenses (inoculants, biochar, chelators), labor, monitoring, and potential revenue streams (e.g., bioenergy conversion, phytomining, or carbon credits). From a regulatory standpoint, naturally occurring PGPR and biochar amendments face fewer deployment barriers, but genetically modified plants, engineered microbial consortia, or synthetic gene circuits must navigate stringent environmental release frameworks that vary widely across jurisdictions [112]. Future field trials should therefore be designed in parallel with regulatory risk assessments, addressing the potential for horizontal gene transfer, non-target ecological effects, and long-term trait stability. Transparent stakeholder engagement, farmer co-design, and policy-aligned validation metrics will be indispensable for transitioning rhizosphere-engineered phytoremediation from academic innovation to scalable soil restoration practice.

### 6.2. Balancing Phytoextraction Efficiency with Food Safety

A critical but often underemphasized tension in Cd phytoremediation research is the risk that interventions designed to maximize Cd accumulation in plant biomass, particularly in crops with edible parts, may inadvertently increase Cd entry into the food chain [102]. Strategies that combine chelator-assisted phytoextraction (e.g., EDTA application) with crop species such as wheat or maize raise particular concerns, as Cd can partition into grain under enhanced uptake conditions. A compelling solution is to confine high-efficiency phytoextraction to non-food species with high biomass production and inherent Cd-accumulation capacity, including *Sedum alfredii*, *Miscanthus sinensis*, *Salix* spp., and hemp (*Cannabis sativa*), while reserving adjacent land for conventional crops with appropriate buffer zones and Cd monitoring [113]. Intercropping designs that alternate hyperaccumulator species with low-Cd-accumulating crops within a plot or rotation offer a pragmatic compromise between remediation efficiency and agricultural productivity and warrant further optimization research.

### 6.3. Regulatory, Biosafety, and Ethical Considerations

Regulatory requirements for rhizosphere-engineered remediation vary by jurisdiction and depend on both the plant and the nature of the modification. Deploying genetically modified microorganisms (GMMs) and transgenic plants in open-field settings for phytoremediation intersects with complex regulatory frameworks that vary substantially across jurisdictions [114]. In the European Union, Directive 2001/18/EC governs the deliberate environmental release of GMMs and requires extensive risk assessment and authorization procedures. In contrast, naturally occurring metal-resistant PGPR strains are not regulated as GMMs provided they have not been genetically modified [115]. Naturally occurring microbial inoculants are not automatically exempt from regulation; depending on the product and jurisdiction, they may be subject to rules governing fertilizers, biostimulants, plant protection products, environmental release, or microbial safety. Furthermore, using engineered gene drives, synthetic metabolic circuits, or CRISPR-edited microbial strains in rhizosphere applications will require new regulatory paradigms that balance innovation with precautionary principles. Biosafety assessments must address horizontal gene transfer of metal resistance determinants, potential non-target ecological effects on indigenous microbial communities and soil fauna, and the stability of engineered traits over multiple generations in field environments [108]. Public engagement and transparent communication of benefits and risks are essential prerequisites for the social license necessary to advance rhizosphere biotechnology from controlled trials to broad agricultural application.

### 6.4. Emerging Technologies for Next-Generation Phytoremediation

Several emerging technology platforms hold substantial promise for transforming the precision and scalability of rhizosphere-engineered phytoremediation. Artificial intelligence and machine learning tools can increasingly analyze large multi-omics datasets from rhizosphere microbiomes to identify non-linear associations between microbial community composition, soil properties, and plant Cd accumulation phenotypes, enabling data-driven microbiome design without requiring a complete mechanistic understanding of individual interactions [99]. Sensor-based soil monitoring technologies, including X-ray fluorescence (XRF) handheld analyzers, mid-infrared spectroscopy probes, and biosensor-based platforms using Cd-responsive microbial reporter strains, are enabling real-time, spatially resolved mapping of Cd distribution and bioavailability at the field scale, allowing precision application of amendments and inoculants where they are most needed. Synthetic biology can engineer custom microbial functions, including Cd-responsive biosensors linked to siderophore biosynthesis actuators or root-colonizing bacteria engineered to constitutively secrete specific organic acids with a level of programmability previously unattainable through natural strain selection [116]. CRISPR-Cas genome editing in both plants and rhizosphere-competent bacteria enables precise, targeted modifications of metal transporter genes, exudate pathway enzymes, and stress tolerance factors without introducing foreign DNA sequences, potentially offering a regulatory pathway distinct from conventional transgenic approaches [117]. Because root exudate composition is species-specific, plant species that host large, Cd-tolerant populations of endophytic and rhizosphere microbes are promising candidates, and future work should prioritize screening for such plant–microbe combinations.

## 7. Conclusions

Cadmium contamination of agricultural soils demands remediation strategies that address not only total metal burden but also the dynamic, plant-available fraction governing crop uptake and food chain transfer. This review has shown that the rhizosphere is the central interface where soil chemistry, root physiology, microbial activity, and intracellular metal handling converge to determine Cd fate. Successful phytoremediation therefore requires deliberate, goal-directed regulation of Cd mobilization, uptake, tolerance, translocation, and sequestration, rather than indiscriminate maximization of metal accumulation.

Three principal conclusions emerge from the evidence presented here. First, microbial groups play functionally distinct, non-interchangeable roles. Plant growth-promoting rhizobacteria enhance short-term Cd mobilization and sustain biomass through phytohormone production and ACC deaminase activity. Arbuscular mycorrhizal fungi promote long-term Cd immobilization through hyphal sequestration and glomalin-associated binding. Endophytes modulate internal metal transport and detoxification within plant tissues. Inoculant selection must therefore be guided by functional traits matched to site-specific remediation goals (phytoextraction versus phytostabilization), rather than by taxonomic identity alone. Second, integrated strategies combining synthetic microbial consortia, targeted soil amendments, and plant genetic approaches yield synergistic gains that exceed the sum of individual interventions, as shown by multi-site field trials with Sedum alfredii and intercropping systems. Third, and most critically, the main bottleneck is no longer mechanistic understanding but translating controlled experiments into persistent field performance. Aged contamination, spatial heterogeneity, seasonal variability, and competition with native soil microbiota systematically erode the efficacy gains observed in pot and greenhouse studies, underscoring the urgent need for multi-season, multi-site validation on historically contaminated soils.

Future progress will depend on adopting standardized field validation protocols with harmonized performance metrics, longitudinally tracking inoculant persistence and microbiome restructuring through multi-omics approaches, and conducting rigorous cost–benefit and food safety assessments alongside regulatory risk analysis. Emerging tools such as artificial intelligence-assisted microbiome design, precision sensing, CRISPR-based genome editing, and synthetic biology offer substantial promise but will ultimately be judged by reproducible field performance, ecological safety, and economic viability.

The future of Cd phytoremediation lies not in identifying a single superior plant, microorganism, or amendment, but in designing rhizosphere systems in which plant genotype, microbial function, soil chemistry, and management practice are deliberately matched to site conditions and remediation goals. By shifting the field toward trait-based design, standardized long-term validation, and simultaneous consideration of food safety, soil health, biosafety, and economic feasibility, rhizosphere engineering can provide a scientifically defensible and scalable pathway for restoring Cd-contaminated agricultural soils.

## Figures and Tables

**Table 1:** Key summary of microbial species and their mechanisms relevant to Cd phytoremediation, discussed in the following subsections.

Microorganisms	Type	Mechanism	Host Plant	References
*Bacillus subtilis*	PGPR	ACC deaminase, siderophore production	*Sedum plumbizincicola*	[44]
*Pseudomonas fluorescens*	PGPR	Exopolysaccharide binding, IAA synthesis	*Triticum aestivum*	[45]
*Rhizophagus irregularis*	AMF	Hyphal immobilization, glomalin production	*Helianthus annuus*	[46]
*Trichoderma asperellum*	Fungal Endophyte	Phytochelatin upregulation, ROS buffering	*Solanum nigrum*	[47]
*Mesorhizobium huakuii*	Endosymbiont	Cd sequestration in nodules, metallothionein expression	*Astragalus sinicus*	[48]
*Glomus mosseae*	AMF	Arbuscular colonization, improved P/Zn nutrition	*Zea mays*	[49]

**Table 2:** Comparison of major rhizosphere engineering strategies, mechanisms, outcomes and limitations.

Strategy	Mechanism	Outcome	Limitation	Reference
Biochar amendment	Sorption of Cd^2+^ onto a high surface-area carbon matrix	Reduced Cd bioavailability; enhanced microbial biomass	Variable with feedstock type	[67]
EDTA chelation	Complexation of Cd to enhance rhizosphere solubility	Increased shoot Cd accumulation >3-fold	Leaching risk; phytotoxicity	[68]
PGPR co-inoculation	Consortium of Bacillus + Pseudomonas strains	Biomass +40%; Cd translocation factor +65%	Strain compatibility issues	[69]
HMA3/ZIP overexpression	Genetic engineering of Cd transporter genes	2–5× increase in Cd in shoots	Regulatory barriers; off-target effects	[70]
Microbiome Transplantation	Inoculation with rhizosphere slurry from hyperaccumulators	Enhanced metal mobilization and plant vigor	Ecological risk; variable outcomes	[71]
Nanoparticle application	nZVI or TiO_2_ NPs to modulate soil chemistry	Altered Cd speciation, improved plant antioxidant capacity	Ecotoxicological risks	[72]

## Data Availability

Data or the source of the data will be made available on request to the first author (T.M.) and (V.E.).
